# Metformin suppresses MEN1-associated pancreatic and pituitary neuroendocrine tumors: evidence from mouse models and clinical data

**DOI:** 10.1530/ERC-25-0518

**Published:** 2026-02-17

**Authors:** Airi Nakano, Yao Huang, Yuri Mistui, Ryunosuke Shirai, Yu Chen, Tomoko Tajima, Yukiko Sakaguchi, Tomoka Moro, Chihiro Hoshino, Kosuke Terada, Naoaki Sakata, Akihiko Yokoyama, Susumu Hijioka, Masamichi Ishiai, Hidetoshi Kassai, Yuko Tabata, Rieko Ohki

**Affiliations:** ^1^ Laboratory of Fundamental Oncology, National Cancer Center Research Institute, Chuo-ku, Tokyo, Japan; ^2^ Graduate School of Biomedical Sciences, Nagasaki University, Nagasaki, Japan; ^3^ Central Animal Division, National Cancer Center Research Institute, Chuo-ku, Tokyo, Japan; ^4^ Tokyo College of Biotechnology, Ohta-ku, Tokyo, Japan; ^5^ Department of Pharmacy, National Cancer Center Hospital, Chuo-ku, Tokyo, Japan; ^6^ Department of Regenerative Medicine and Transplantation, Faculty of Medicine, Fukuoka University, Fukuoka, Japan; ^7^ National Cancer Center Tsuruoka Metabolomics Laboratory, Yamagata, Japan; ^8^ Department of Hepatobiliary and Pancreatic Oncology, National Cancer Center Hospital, Tokyo, Japan; ^9^ Central Radioisotope Division, National Cancer Center Research Institute, Chuo-ku, Tokyo, Japan

**Keywords:** pancreatic neuroendocrine tumor, MEN1, metformin, PI3K/Akt/mTOR pathway, glucose control, pituitary NET

## Abstract

Pancreatic neuroendocrine tumors (PanNETs) represent a rare subset of pancreatic cancers, comprising approximately 1–2% of all cases. Non-functioning PanNETs (NF-PanNETs), which account for the majority of PanNETs, can be difficult to treat as they show no hormone-related symptoms and are often not diagnosed until more advanced stages. Current therapeutic agents have limited efficacy, highlighting the need for novel treatment strategies. Multiple endocrine neoplasia type 1 is a hereditary syndrome strongly associated with PanNETs and pituitary neuroendocrine tumors (PitNETs), caused by germline mutations in the *MEN1* gene. Using *Men1*^f/f−^RipCre^+^ mice, which develop both NF-PanNETs and PitNETs, we investigated whether long-term administration of metformin, a first-line anti-diabetic drug, could suppress tumor development and progression. Metformin significantly inhibited the elevation of blood glucose in *Men1*^f/f−^RipCre^+^ mice, and longer-term treatment attenuated PanNETs and restored normal insulin secretion. Metformin suppressed the proliferative pathways, including the PI3K/Akt/mTOR signaling pathway. In addition to its effects on PanNETs, metformin also attenuated PitNET development, elevated antiproliferative pathways, and suppressed angiogenic pathways. Furthermore, clinical data revealed that NF-PanNET patients with prior metformin use exhibited improved prognosis. These findings demonstrate that blood glucose control through metformin represents a promising preventive and therapeutic strategy for NF-PanNETs and MEN1-associated neuroendocrine tumors.

## Introduction

Pancreatic islets are endocrine tissues that secrete hormones such as insulin and glucagon, which are essential for glucose homeostasis. Pancreatic neuroendocrine tumors (PanNETs) mainly originate from these endocrine cells. Although PanNETs account for only 1–2% of all pancreatic cancers, non-functioning PanNETs (NF-PanNETs), which lack hormone-related symptoms, are frequently diagnosed at advanced stages and have a poor prognosis, with a five-year survival rate of approximately 40%. Because metastatic cases are often unresectable and current systemic therapies show limited efficacy, new treatment strategies are urgently needed.

Neuroendocrine tumors occur at a high frequency in hereditary syndromes, particularly multiple endocrine neoplasia type 1 (MEN1). A diagnosis of MEN1 is based on one of the following: a pathogenic MEN1 germline mutation, a positive family history, or the occurrence of two or more MEN1-related tumors. Experimental studies on MEN1-related NETs frequently utilize *Men1*^f/f−^RipCre^+^ mice (1). While *Men1* deletion targeting exons 3–8 or exon 3 leads to functional insulinomas, we previously generated *Men1*^f/f−^RipCre^+^ mice lacking exons 3–6, which reproducibly develop both NF-PanNETs and PitNETs (prolactinomas) by 45 weeks of age, closely recapitulating tumors in human MEN1 patients (2).

Pancreatic β-cell proliferation is regulated by the insulin–PI3K/Akt–mTOR signaling pathway, which is frequently hyperactivated in human PanNETs (3, 4, 5). Our previous studies demonstrated that loss of PHLDA3, a physiological Akt inhibitor, activates this pathway and promotes islet hyperplasia in mice and PanNET progression in humans (4, 5, 6, 7, 8). We also found that a ketogenic diet reduces insulin secretion, suppresses PI3K/Akt activation, and prevents NF-PanNETs development in *Men1*^f/f−^RipCre^+^ mice (9). However, long-term adherence to ketogenic diets is often difficult because of dietary restrictions and associated metabolic burden, prompting the need for simpler alternatives (10). We hypothesized that metformin, a widely used type 2 diabetes drug, could suppress NF-PanNET progression by lowering blood glucose and inhibiting insulin–PI3K/Akt–mTOR signaling.

Hyperinsulinemia and hyperglycemia in type 2 diabetes increase the risk of various cancers, including breast, colorectal, and pancreatic cancers (11, 12, 13, 14, 15, 16), and elevated insulin levels correlate with cancer susceptibility (17, 18). PanNET patients also frequently develop diabetes, further implicating insulin signaling in tumor progression (19, 20). Clinical reports indicate that diabetic patients treated with metformin have lower pancreatic cancer risk and mortality (21, 22, 23, 24, 25, 26). Given its long-term safety (26, 27), we considered that metformin may also be effective in suppressing NF-PanNETs and pituitary tumors in MEN1 models.

In this study, we investigated the impact of long-term metformin administration in Men1^f/f−^RipCre^+^ mice. Metformin inhibited tumor development while preserving islet and pituitary function in association with suppression of PI3K/Akt–mTOR signaling. Consistently, NF-PanNET patients receiving metformin exhibited a prolonged overall survival (OS). These findings support metformin as a practical preventive or therapeutic option for MEN1-associated and sporadic neuroendocrine tumors.

## Materials and methods

### Mice used in this study

All mouse experiments were conducted in a specific pathogen-free facility at the National Cancer Center in accordance with institutional guidelines. All procedures were approved by the Committee for Ethics in Animal Experimentation at the National Cancer Center (protocol numbers: T17-002-C01 and T17-011-M03). The general animal housing conditions and genetic background of the *Men1*^f/f−^RipCre^+^ (exons 3–6 floxed) mice have been previously described (9). The *Men1*^f/f−^RipCre^+^ mice develop both pancreatic neuroendocrine tumors (PanNETs) and pituitary neuroendocrine tumors (PitNETs). By 45 weeks of age, most mice develop PanNETs, and the majority of female mice additionally develop PitNETs. These mice were therefore used to assess the long-term effects of metformin on tumor development. Wild-type control mice were C57BL/6. Mice were maintained on a standard chow diet consisting of 49.8% carbohydrate, 25.1% protein, 4.8% fat, 4.4% fiber, and 7.0% ash with minerals and vitamins (CE-2, CLEA Japan, Japan) with water access *ad libitum*. For experimental groups, the MEN1-NT cohort received regular drinking water, whereas the MEN1-met cohort received water containing metformin hydrochloride (500 mg, MT ‘TE’; TOA EIYO Ltd, Japan) at a final concentration of 5 mg/mL, administered orally from 10 to 45 weeks of age.

### Blood glucose measurement

Blood samples were collected between 13:00 and 16:00 h from the tail vein. Random blood glucose levels were determined using a Glucose Pilot instrument (Aventir Biotech, USA), whereas glucose levels during GTTs were measured using the AllChek II Blood Glucose Meter with compatible sensor devices and test strips (Hangzhou AllTest Biotech Co., Ltd, China), according to the manufacturers’ protocols.

### Immunohistochemistry (IHC)

IHC was performed as previously described (4). In brief, paraffin-embedded sections were deparaffinized and subjected to antigen retrieval in 10 mM citrate buffer (pH 6.0) by autoclaving. Endogenous peroxidase activity was quenched using 3% H_2_O_2_, and nonspecific binding was blocked for 1 h in 5% goat serum.

Primary antibodies included rabbit anti-Ki67 monoclonal antibody (SP6, Novus Biologicals, USA; 1:100), rabbit anti-p21 (sc-397, Santa Cruz, USA; 1:100), rabbit anti-P-S6 (#5364, Cell Signaling Technology, USA; 1:1,000), rabbit anti-P-AMPK (#2535, Cell Signaling, USA; 1:100), rabbit anti-rat prolactin (Institute for Molecular and Cellular Regulation, Gunma University; 1:10,000), and rabbit anti-rat TSH (Institute for Molecular and Cellular Regulation, Gunma University; 1:5,000). Secondary antibodies included biotinylated anti-rabbit IgG and anti-mouse IgG (VECTOR Laboratories, USA; 1:500). SignalStain Boost Detection Reagent (HRP, Rabbit #8114, Cell Signaling Technology, USA) was also used as appropriate. Staining was visualized using 3,3′-diaminobenzidine tetrahydrochloride (DAB; Muto Pure Chemicals, Japan) and counterstained with hematoxylin.

For fluorescent IHC of insulin, sections were blocked with 5% goat serum, followed by incubation with rabbit anti-insulin monoclonal antibody (#19H4L12, Invitrogen, USA; 1:300, with Signal Enhancer HIKARI, NACALAI TESQUE, Japan) overnight at 4°C. Alexa Fluor 594 goat anti-rabbit IgG (A11037, Invitrogen, USA; 1:1,000) was used as a secondary antibody. Slides were counterstained with DAPI.

### Isolation of mouse islets and NETs

Isolation of mouse islets and NETs was performed as previously described (4). In brief, mouse islets and NETs were isolated from 45-week-old animals by collagenase digestion of the pancreas, followed by purification in a Ficoll gradient. Islets and NETs were handpicked and used for the subsequent analysis.

### Western blotting analysis

Cells lysis buffer was 50 mM Tris–HCl (pH 8.0), 1% NP40, 250 mM NaCl, 1 mM DTT, 5 mM EDTA, and 1 mM protease inhibitor (PMSF, aprotinin, and leupeptin). The lysates were then treated with an equal volume of 4X SDS buffer (0.4 M Tris–HCl (pH 6.8), 8% SDS, 4% (v/v) glycerol, and 0.04% bromophenol blue). To detect anti-β-actin and AMPK, 10 μg whole cell lysates obtained from isolated islets and NETs were loaded. Antibodies used in this study are rabbit anti-P-AMPK (#2535, Cell Signaling, USA; 1:1,000) and mouse anti-β-actin (MAB1501, Millipore, USA; 1:10,000).

### Reverse transcription and real-time PCR

Total RNA was extracted using the AllPrep DNA/RNA/Protein mini kit (Qiagen, UK). Reverse transcription was performed with the SuperScript First-Strand Synthesis System for RT-PCR (Invitrogen, USA). Real-time PCR was carried out using SYBR Green Premix Ex Taq (Tli RNaseH Plus, Takara Bio Inc., Japan) on a CFX Duet Real-Time PCR System (Bio-Rad, UK).

TaqMan probe for mouse 18S rRNA from Integrated DNA Technologies and custom-designed primers were used for the SYBR green system to detect gene expression. Relative mRNA expression was normalized to 18S ribosomal RNA.

Primer sequences included the following:

*Bcl6* (F: CAG​AGA​TGT​GCC​TCC​ATA​CTG​C; R: CTC​CTC​AGA​GAA​ACG​GCA​GTC​A), *Ccnd1* (F: GCA​GAA​GGA​GAG​ATT​GTG​CCA​T; R: AGG​AAG​CGG​TCC​AGG​TAG​TTC​A), *ChgA* (F: AGC​ATC​CAG​TTC​CCA​CTT​C; R: CTC​TGT​CTT​TCC​ATC​TCC​ATC​C), *Pnlip* (F: ACC​GAT​GCT​CAG​TTT​GTG​GAC​G; R: CCT​GGC​ATT​TCG​ATT​CCT​CCG​T), *p21* (F: AAT​CTG​CGC​TTG​GAG​TGA​TAG; R: CTT​GTC​GCT​GTC​TTG​CAC​T), *Cdkn1b* (F: AGC​AGT​GTC​CAG​GGA​TGA​GGA​A; R: TTC​TTG​GGC​GTC​TGC​TCC​ACA​G), *Mmp2* (F: CAA​GGA​TGG​ACT​CCT​GGC​ACA​T; R: TAC​TCG​CCA​TCA​GCG​TTC​CCA​T), *Mmp9* (F: GCT​GAC​TAC​GAT​AAG​GAC​GGC​A; R: TAG​TGG​TGC​AGG​CAG​AGT​AGG​A), *Nos2* (F: GAG​ACA​GGG​AAG​TCT​GAA​GCA​C; R: CCA​GCA​GTA​GTT​GCT​CCT​CTT​C), *Igfbp7 *(F: AAG​AGG​CGG​AAG​GGT​AAA​GC; R: TGG​GGT​AGG​TGA​TGC​CGT​T), and *Eif4a1 *(F: TCA​TGT​CTG​CGA​GTC​AGG​AT; R: GCT​ATC​CAC​AAT​CTC​GTT​CCA).

### Quantitative measurement of islet and pituitary morphology

Islet area, nuclear number, and pituitary area were measured from hematoxylin–eosin-stained sections. DAB-based immunohistochemistry images were analyzed semi-quantitatively as previously described (28). The Ki67 labeling index was calculated as the proportion of positive cells in the region with the highest staining intensity at ×200 magnification. For each pancreas, images of five representative islets were captured. Insulin, p21, P-S6, and P-AMPK expression levels were quantified from five representative islets per pancreas. For MEN1 mice, islets larger than 0.01 mm^2^ were analyzed, excluding normal islets. Analyses were conducted on samples from WT (*n* = 3), MEN1-NT (*n* = 3), and MEN1-met (*n* = 4) mice using a BZ-X810 microscope and software (Keyence). P-S6 and P-AMPK were measured in mice that underwent a GTT (MEN1-NT: *n* = 3 and MEN1-met: *n* = 3). For presentation, fluorescent image brightness was adjusted uniformly in Photoshop (Adobe Systems), while quantitative analyses were performed using unmodified data.

### Patients analyzed in the study

A retrospective cohort study was conducted, which consisted of 61 patients with pathologically confirmed grade 2 NF-PanNETs who were 50–71 years of ages and who had undergone chemotherapy without surgery between August 2010 and March 2022 at the National Cancer Center Hospital. Chemotherapies included octreotide, lanreotide, everolimus, sunitinib, lanreotide + everolimus, lanreotide + sunitinib, and streptozotocin + 5-FU. The inclusion criteria were as follows: i) patients diagnosed with grade 2 NF-PanNET, ii) patients aged 50–71 years, iii) patients that had follow-up data, iv) no prior chemotherapy before the chemotherapy analyzed in this study, and v) past surgical resection only if performed ≥1 year before the initiation of the chemotherapy. We excluded cases where i) patients had been diagnosed with functional PanNETs or ii) patients had developed multiple primary tumors. This study was approved by the Institutional Review Board of the National Cancer Center, Tokyo (approval number: 2013-023). Informed consent was obtained for all cases. Clinical and pathological data were obtained through a detailed retrospective review of the medical records of all PanNET patients. OS was defined as the length of time (in days) from the beginning of treatment to death from any cause.

### Statistical analysis

Data were calculated and are shown as mean ± SEM. Comparisons between the samples were performed by Student’s *t* test or by one-way ANOVA multiple comparisons (Tukey’s multiple comparisons test) using Prism software (version 10). For the *t* test, Student’s *t* test was used when the variances of the groups were not significantly different, as evaluated by F-test. Survival data were analyzed using Prism software (version 10), and Kaplan–Meier plots were drawn. Statistical significance was defined as *P* < 0.05. For animal studies, no statistical methods were used for sample size estimates, no samples were excluded, no randomization was used, and no blinding was done. For the human studies shown in Fig. 5, no statistical methods were used to estimate sample size.

## Results

### Metformin administration suppresses blood glucose elevation in wild-type and *Men1*^f/f−^RipCre^+^ mice

To determine whether metformin alters dietary intake, fluid consumption, and/or caloric uptake in wild-type (WT) mice, female WT mice (10–12 weeks old) were given either regular drinking water or water containing 5 mg/mL metformin. We saw no significant differences in the daily food intake, water intake, or caloric consumption between the two groups (Supplementary Fig. S1A, S1B, S1C (see section on Supplementary materials given at the end of the article)). Based on water intake, the estimated daily metformin dose exceeded 1,000 mg/kg (Supplementary Fig. S1D). For reference, the clinical dose in type 2 diabetic patients is 500–2,550 mg/day, equivalent to ∼35 mg/kg/day (29). Previous murine studies demonstrated that more than 50–250 mg/kg metformin will reduce blood glucose levels and suppress tumorigenesis (30, 31, 32). Thus, our administration protocol provided a supratherapeutic dose relative to the reported effective ranges in mice.

Next, we examined whether metformin suppressed glucose elevation in *Men1*^f/f−^RipCre^+^ mice. Mice (11–12 weeks old) were subjected to glucose tolerance tests (GTTs) following a 48 h fast. Mice were administered metformin-containing water (5 mg/mL) for 6 h *ad libitum*. After that, mice were given intraperitoneal glucose (2.0 g/kg body weight), and blood glucose was measured 30 min later. Metformin significantly suppressed glucose elevation compared with untreated controls (Supplementary Fig. S1E and S1F). Collectively, these results demonstrate that oral metformin at 5 mg/mL is well tolerated, does not impair dietary intake, and reduces blood glucose levels, thereby suppressing postprandial hyperglycemia in mice.

### Long-term metformin administration suppresses PanNET progression in *Men1*^f/f−^RipCre^+^ mice and restores islet function

**Figure 1:**
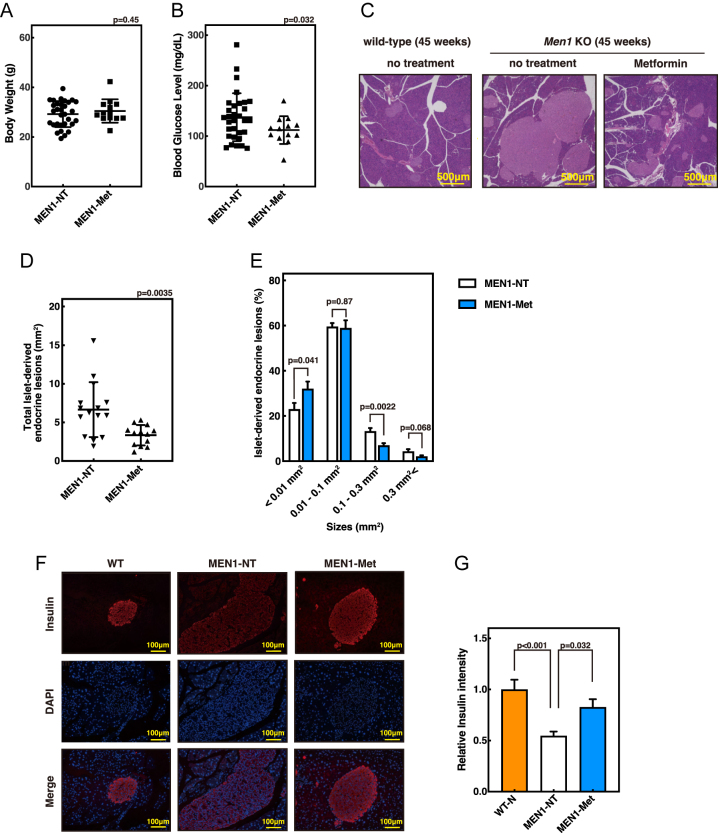
Long-term metformin administration suppresses PanNET progression in *Men1*^f/f−^RipCre^+^ mice. Female *Men1*^f/f−^RipCre^+^ mice were untreated (MEN1-NT, *n* = 31) or administered 5 mg/mL metformin from 12 to 14 weeks of age until sacrifice at 45 weeks *ad libitum* (MEN1-met, *n* = 13). (A and B) Body weight (A) and random blood glucose (B) at 45 weeks. (C) Representative hematoxylin–eosin (H&E) staining of pancreatic sections. (D and E) Quantification of islet area (MEN1-NT, *n* = 15; MEN1-met, *n* = 13) (D) and comparison of islet size categories (E). (F and G) Insulin immunofluorescence (F) and quantification of fluorescence intensity (G). For each group, three mice whose total islet area was closest to the group mean were analyzed, with five islets imaged per mouse. For MEN1 mice, islets larger than 0.01 mm^2^ were analyzed, excluding normal islets. Analyses were performed on samples from WT (*n* = 3), MEN1-NT (*n* = 3), and MEN1-met (*n* = 4) mice. A full-colour version of this figure is available at https://doi.org/10.1530/ERC-25-0518.

We previously demonstrated that *Men1*^f/f−^RipCre^+^ mice exhibit preneoplastic lesions at 30 weeks of age and develop pituitary and pancreatic β-cell neuroendocrine tumors by 45 weeks (9). Early dietary intervention with a ketogenic regimen from 10 weeks of age suppressed high blood glucose levels and inhibited tumor onset and progression. Given that metformin has been reported to suppress tumor growth in various cancer models (30, 31, 32), we investigated whether long-term administration of metformin could similarly attenuate PanNET development in *Men1*^f/f−^RipCre^+^ mice. Mice were provided with drinking water containing 5 mg/mL metformin from ∼10 weeks of age until 45 weeks, at which point they were sacrificed. Body weight did not differ between metformin-treated and untreated mice (Fig. 1A). However, random blood glucose levels were significantly reduced in metformin-treated animals compared with controls (Fig. 1B). Histological examination of hematoxylin–eosin (H&E)-stained pancreatic sections revealed a significant reduction in islet area in the metformin-treated group, indicating suppression of PanNET progression (Fig. 1C and D). To further characterize islet morphology, islets were classified according to their area, based on our previous report (4), as normal (<0.01 mm^2^), hyperplastic (0.01–0.1 mm^2^), PanNET (0.1–0.3 mm^2^), or large PanNET (>0.3 mm^2^), and their distribution was analyzed. Metformin treatment resulted in an increased proportion of normal islets and a significantly decreased proportion of PanNETs (Fig. 1E).

We previously showed that by 45 weeks of age, *Men1*^f/f−^RipCre^+^ mice exhibit markedly reduced, abnormal insulin staining in PanNETs, indicative of impaired hormone secretion in non-functioning tumors (9). Consistent with this, untreated *Men1*^f/f−^RipCre^+^ mice in the present study displayed diminished insulin immunoreactivity compared with wild-type controls. On the other hand, long-term metformin treatment restored insulin staining intensity, indicating recovery of hormone secretion ability in β-cells (Fig. 1F and G). Long-term metformin treatment reduces overall NF-PanNET size in Men1^f/f−^RipCre^+^ mice, and the NF-PanNET cells regain β-cell-like function and insulin production. Consequently, insulin content per cell is normalized, resulting in a relatively stronger insulin signal despite the smaller size of insulin-positive lesions compared with non-treated Men1^f/f−^RipCre^+^ NF-PanNETs.

Together, these results demonstrate that long-term oral administration of metformin significantly suppresses the progression of non-functioning PanNETs in *Men1*^f/f−^RipCre^+^ mice, restores insulin expression, and thereby improves islet function.

### Metformin suppresses PanNET cell proliferation and restores p21 expression in *Men1*^f/f−^RipCre^+^ mice

**Figure 2:**
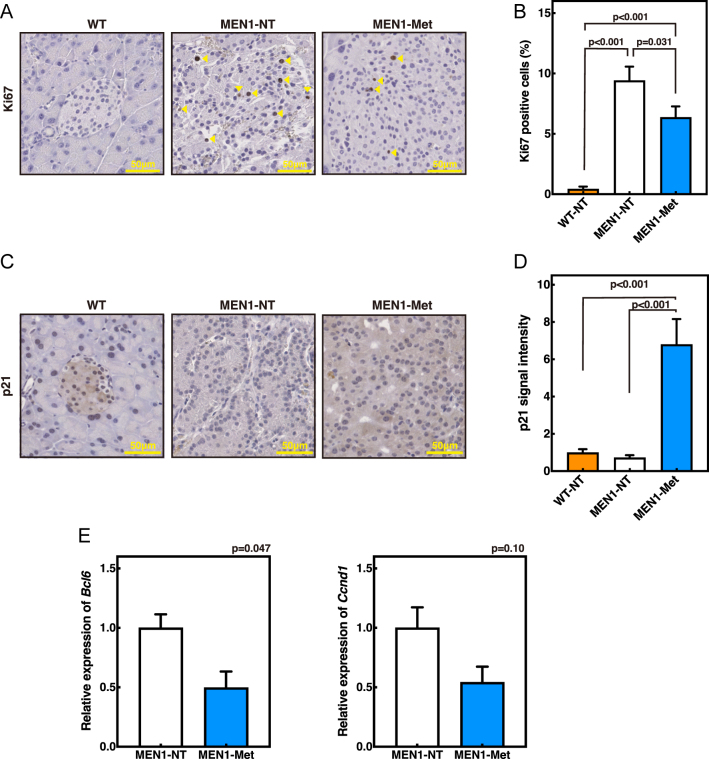
Metformin suppresses cell proliferation in PanNETs of *Men1*^f/f−^RipCre^+^ mice. (A and B) Ki67 immunostaining (A) and quantification of Ki67-positive cell ratios in pancreatic sections from 45-week-old mice (B). Five islets per mouse were analyzed, and within each islet, the region with the highest Ki67 labeling index was quantified. WT (*n* = 3), MEN1-NT (*n* = 3), and MEN1-met (*n* = 4). (C and D) p21 immunostaining (C) and quantification of signal intensity (D). Five islets per mouse were analyzed. For MEN1 mice, islets larger than 0.01 mm^2^ were analyzed, excluding normal islets. Analyses were conducted on samples from WT (*n* = 3), MEN1-NT (*n* = 3), and MEN1-met (*n* = 4) mice. (E) Gene expression analysis of proliferation-related genes (*Ccnd1* and *Bcl6*) in isolated islets and NETs from 45-week-old male mice. MEN1-NT (*n* = 3 replicates) and MEN1-met (*n* = 3 replicates). Islets were pooled from multiple mice (MEN1-NT: *n* = 3, 2, 1; MEN1-met: *n* = 5, 5, 2 per replicate). A full-colour version of this figure is available at https://doi.org/10.1530/ERC-25-0518.

To determine whether the reduction in PanNET size by metformin was attributable to the suppression of cell proliferation, we examined Ki67 expression, a marker of proliferating cells, by immunochemistry. As average islet size varied among groups, we took 7–10 islets per pancreas that approximated the average size for each group. Of these, the three islets exhibiting the highest Ki67 labeling index were analyzed, and regions with maximal positivity were quantified. In untreated 45-week-old *Men1*^f/f−^RipCre^+^ mice, the proportion of Ki67-positive cells was markedly higher compared with wild-type controls. Importantly, metformin treatment significantly reduced the fraction of Ki67-positive cells (Fig. 2A and B), demonstrating that metformin suppresses islet cell proliferation *in vivo*.

Next, we examined p21, a cyclin-dependent kinase inhibitor known to restrain cell cycle progression. Previous studies have shown that *p21*-deficient mice exhibit impaired glucose homeostasis and increased susceptibility to type 2 diabetes, suggesting an important role for p21 in pancreatic islet regulation (33). In untreated *Men1*^f/f−^RipCre^+^ mice, p21 expression in islets was comparable to that in wild-type controls. Notably, metformin significantly increased p21 expression, suggesting that metformin not only suppresses proliferation but also induces p21-mediated cell cycle control in PanNETs (Fig. 2C and D).

To further confirm these findings, we performed gene expression analysis of isolated pancreatic islets and NETs. Islets and NETs were pooled from multiple mice (MEN1-NT (non-treated): *n* = 3, 2, 1 mice per replicate; MEN1-metformin: *n* = 5, 5, 2 mice per replicate) and analyzed by quantitative RT-PCR. We confirmed the purity of the isolated islets and NETs by evaluating the transcript levels of *Chga* (endocrine marker) and *Pnlip* (exocrine marker) (Supplementary Fig. S2A and S2B). Metformin treatment reduced the expression of proliferation-associated genes, including a significant decrease in *Bcl6* and a decreasing trend in *Ccnd1* compared with untreated controls (Fig. 2E).

Together, these findings demonstrate that metformin suppresses tumor progression in *Men1*^f/f−^RipCre^+^ mice by reducing islet cell proliferation, restoring p21 expression, and downregulating proliferative gene programs, thereby reinforcing cell cycle control in non-functioning PanNETs.

### Metformin suppresses PI3K/Akt–mTOR signaling in *Men1*^f/f−^RipCre^+^ PanNETs

Pancreatic islet proliferation is primarily regulated by the insulin–PI3K/Akt–mTOR pathway, which is frequently hyperactivated in human PanNETs (3, 4, 5). We previously showed that dietary intervention with a ketogenic regimen suppresses insulin secretion, thereby inhibiting PI3K/Akt signaling and reducing NF-PanNET development in *Men1*^f/f−^RipCre^+^ mice (9). To test whether metformin exerts similar effects, we examined its impact on the PI3K/Akt–mTOR pathway in PanNETs.

**Figure 3:**
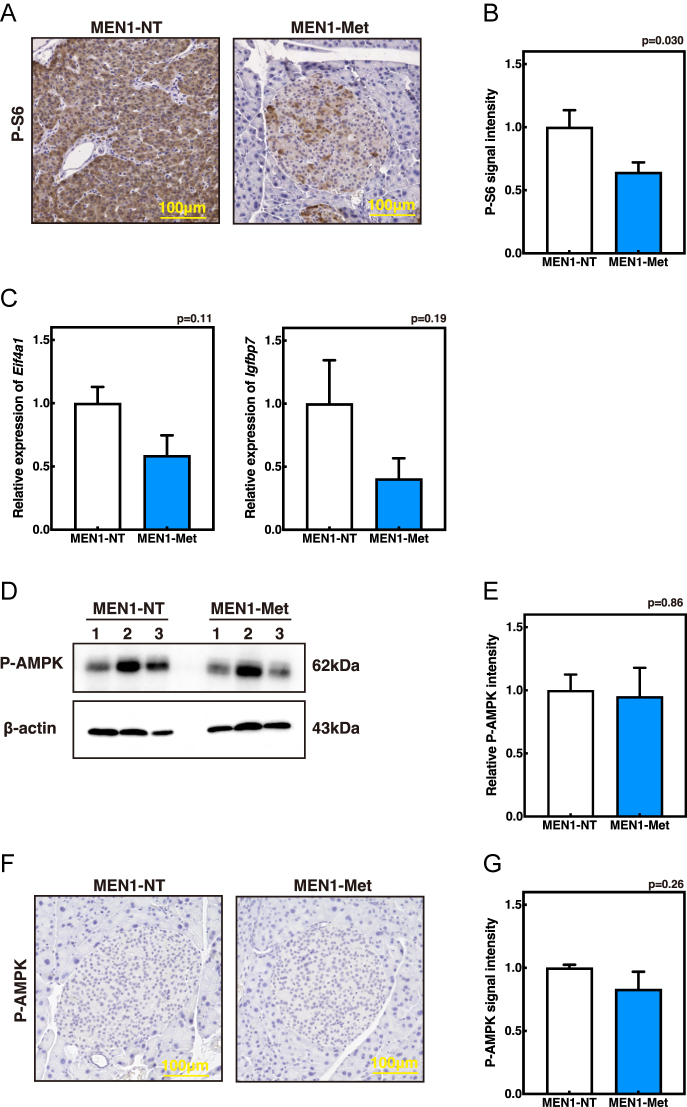
Metformin suppresses PI3K/Akt–mTOR signaling in PanNETs from *Men1*^f/f−^RipCre^+^ mice. (A and B) P-S6 immunostaining (A) and quantification of P-S6-positive area (B) in pancreatic islets. Mice (30–34 weeks old) were fasted for 48 h, including the final 16 h without water, followed by 6 h of access to water containing metformin (5 mg/mL) or regular water *ad libitum*, and intraperitoneal injection of glucose (2.0 g/kg body weight). Pancreata were collected 30 min later. Three islets larger than 0.01 mm^2^ per mouse were analyzed. (C) Gene expression analysis of Akt downstream targets (*Igfbp7* and *Eif4a1*) in isolated islets and NETs from 45-week-old male mice. MEN1-NT: untreated (*n* = 3). MEN1-met: metformin-treated from 8 to 14 weeks until 45 weeks (*n* = 3). Islets were pooled from multiple mice as described in Fig. 2E. (D and E) Western blotting (D) and quantification of P-AMPK band intensities (E) in isolated islets and NETs from 45-week-old male mice. MEN1-NT: untreated (*n* = 3). MEN1-met: metformin-treated from 8 to 14 weeks until 45 weeks (*n* = 3). Western blotting was repeated three times, and the mean P-AMPK levels are shown. Islets were pooled from multiple mice as described in Fig. 2E. (F and G) Immunohistochemistry (F) and quantification of P-AMPK intensities (G) in islets and NETs. The same mice analyzed in Fig. 3A and B were used. Three islets larger than 0.01 mm^2^ per mouse were analyzed. Three islets larger than 0.01 mm^2^ per mouse were analyzed. A full-colour version of this figure is available at https://doi.org/10.1530/ERC-25-0518.

Phosphorylation of ribosomal protein S6 (P-S6), a downstream readout of mTOR activity, was assessed by immunohistochemistry. *Men1*^f/f−^RipCre^+^ mice at 30 weeks of age were subjected to a GTT, and pancreas were harvested 30 min after glucose injection, when blood glucose levels exhibited maximal divergence between control and metformin-treated groups. Importantly, metformin administration significantly reduced the proportion of P-S6-positive cells per islet, indicating suppression of mTOR pathway activity within PanNETs (Fig. 3A and B). We further validated these findings through gene expression profiling of isolated islets and NETs. Islets were pooled from multiple mice (MEN1-N: *n* = 3, 2, 1 per replicate; MEN1-met: *n* = 5, 5, 2 per replicate) and subsequently analyzed by western blotting and qPCR. In line with the reduced p-S6 signal, the expression of Akt pathway-related proliferative genes, including *Eif4a1* and *Igfbp7*, showed a decreasing trend in metformin-treated samples (Fig. 3C).

Metformin has been reported to suppress mTOR signaling and inhibit tumor cell proliferation in several cancers through AMPK activation (34, 35). To examine whether this mechanism also contributes to PanNET suppression in our model, we quantified p-AMPK levels by western blotting and immunohistochemistry. However, as shown in Fig. 3D, E, F, G, p-AMPK levels were comparable between metformin-treated and untreated groups, suggesting that metformin suppresses mTOR signaling and PanNET development predominantly through an AMPK-independent mechanism. This interpretation is consistent with previous reports demonstrating that metformin can inhibit mTOR signaling even in AMPK-deficient cells (34).

Together, these results demonstrate that metformin effectively suppresses the PI3K/Akt–mTOR signaling cascade in *Men1*^f/f−^RipCre^+^ islets, thereby inhibiting the proliferative signaling that drives non-functioning PanNET progression.

### Metformin suppresses PitNET progression in *Men1*^f/f−^RipCre^+^ mice

**Figure 4:**
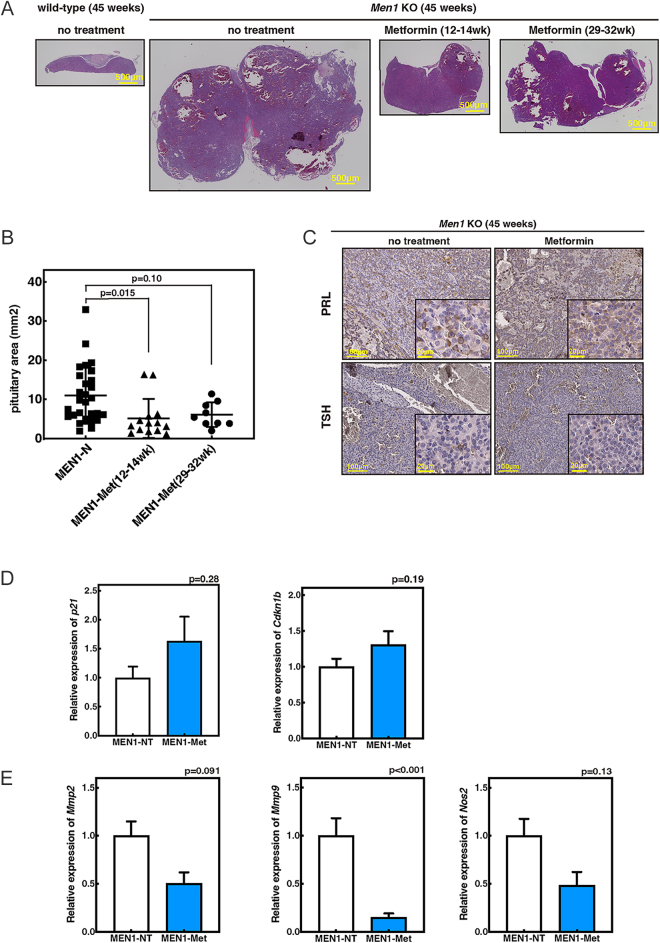
Metformin suppresses PitNET progression in *Men1*^f/f−^RipCre^+^ mice. (A and B) Representative H&E staining (A) and quantification of pituitary area in 45-week-old female mice (B). MEN1-NT (*n* = 28), MEN1-met (metformin from 12 to 14 weeks, *n* = 14), and MEN1-met (metformin from 29 to 32 weeks, *n* = 9). (C) Immunostaining for prolactin (PRL) and TSH in pituitary tumors. (D and E) Gene expression of p53 target genes (D) and angiogenesis-related (*Mmp9* and *Mmp2*) and inflammation-related (*Nos2*) genes (E) in pituitary tissues from tumor-bearing mice. MEN1-NT: *n* = 12; MEN1-met: *n* = 4. A full-colour version of this figure is available at https://doi.org/10.1530/ERC-25-0518.

In *Men1*^f/f−^RipCre^+^ mice, pituitary neuroendocrine tumors (PitNETs) develop with high penetrance, particularly in females (9). We therefore examined whether metformin, in addition to suppressing PanNETs, could also inhibit PitNET progression. Female *Men1*^f/f−^RipCre^+^ mice were divided into three groups: untreated controls maintained on regular water, mice receiving metformin from 12 to 14 weeks of age (prior to tumor onset), and mice receiving metformin from 29 to 32 weeks of age (after preneoplastic lesion formation). All groups were sacrificed at 45 weeks of age. Histological analysis showed a significant enlargement of the pituitary area in untreated versus treated mice, suggesting greater tumor development. Importantly, early metformin administration significantly suppressed this pituitary enlargement, indicating that metformin can restrain pituitary NET development (Fig. 4A and B). In addition, late metformin administration also had a tendency to suppress pituitary enlargement after the onset of preneoplastic changes (Fig. 4A and B). To further characterize the nature of these tumors, we performed immunostaining for prolactin (PRL) and thyroid-stimulating hormone (TSH). Both untreated and metformin-treated PitNETs expressed PRL, whereas TSH expression was negligible (Fig. 4C). These results indicate that *Men1*^f/f−^RipCre^+^ PitNETs are primarily prolactinomas and that metformin effectively suppresses their progression.

Gene expression profiling of pituitary tissues from tumor-bearing mice provided additional mechanistic insights. Notably, metformin treatment showed a trend toward increased expression of cell cycle regulators, such as *p21* and *Cdkn1b* (not statistically significant), while significantly reducing the expression of the angiogenesis-related gene *Mmp9* and showing decreasing trends in *Mmp2* and the inflammation-associated gene *Nos2* (Fig. 4D and E). These results suggest that metformin inhibits PitNET progression not only by direct regulation of the cell cycle but also by modulating the tumor microenvironment through alterations in angiogenesis and inflammatory signaling.

Taken together, these findings demonstrate that metformin suppresses the progression of prolactin-producing PitNETs in *Men1*^f/f−^RipCre^+^ mice by modulating both the intrinsic cell cycle and the extrinsic microenvironment.

### Metformin use is associated with improved prognosis in patients with non-functioning PanNETs

To evaluate the clinical relevance of our findings in mice, we next analyzed the association between the use of blood glucose-lowering agents, metformin, SGLT inhibitors, or voglibose and OS in patients clinically diagnosed with non-functioning PanNETs who had received chemotherapy. As non-functional PanNETs are rare tumors, the number of patients treated with metformin alone in our cohort was very limited, making it difficult to perform a survival analysis. For this reason, we also included patients who had received other glucose-lowering agents, such as SGLT inhibitors or voglibose, which are clinically used anti-diabetic drugs, in order to increase the sample size for exploratory statistical evaluation. The patient age was restricted to 50–71 years, tumor grade was limited to grade 2, and patients with multiple primary tumors were excluded, minimizing biological variability in both tumor aggressiveness and age-related comorbidities. These restrictions were applied because all patients in the blood glucose-lowering agents-treated group had grade 2 tumors, and the age range was chosen to match the ages of patients with or without blood glucose-lowering agents. In total, 61 patients were included, comprising 45 in the control group and 16 in the metformin group.

**Figure 5:**
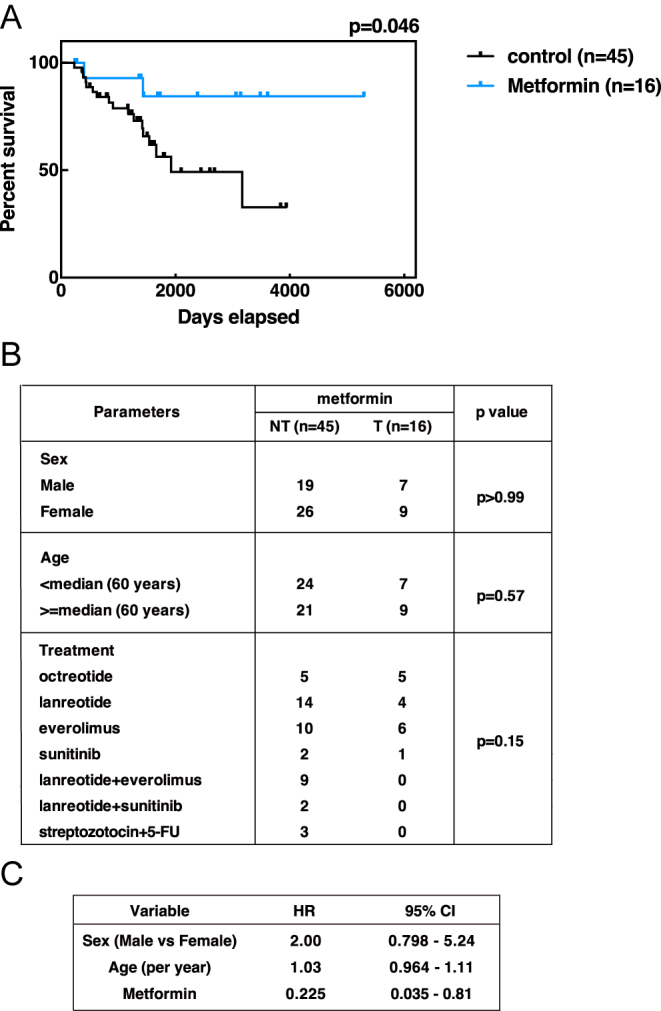
Metformin use is associated with improved prognosis in NF-PanNET patients. (A) Kaplan–Meier analysis of OS in grade 2 NF-PanNET patients aged 50–71 years, with or without a history of use of blood glucose-lowering agents, metformin, SGLT inhibitors, or voglibose. Control group: *n* = 45; blood glucose-lowering agents group: *n* = 16. Patients with multiple primary tumors were excluded. Blood glucose-lowering agent users exhibited a significantly improved survival (*P* = 0.046). (B) Clinicopathological features of control and blood glucose-lowering agent use groups. (C) Cox proportional hazards analysis of OS in grade 2 NF-PanNET patients aged 50–71 years, evaluating the effect of blood glucose-lowering agent use. HRs with 95% confidence intervals (CIs) are shown. A full-colour version of this figure is available at https://doi.org/10.1530/ERC-25-0518.

Kaplan–Meier survival analysis revealed that patients with a history of metformin use had a significantly improved OS compared with controls (*P* = 0.046; Fig. 5A). Importantly, no other significant differences were observed between these two groups with respect to other major clinicopathological features, including sex and age (Fig. 5B). Furthermore, in Cox proportional hazards analysis – adjusting for age, sex, and other relevant clinicopathological covariates – metformin use remained independently associated with an improved OS (hazard ratio (HR) = 0.225, 95% confidence interval = 0.035–0.81) (Fig. 5C).

Collectively, these findings suggest that metformin use is associated with a favorable prognosis in patients with non-functioning PanNETs, consistent with our preclinical data indicating that metformin can suppress tumor progression.

## Discussion

In this study, we demonstrated that long-term administration of metformin, an anti-diabetic drug that suppresses elevation of blood glucose levels, significantly inhibited the progression of non-functioning pancreatic neuroendocrine tumors (NF-PanNETs), as well as pituitary NETs (PitNETs) in *Men1*^f/f−^RipCre^+^ mice.

NF-PanNETs represent the majority of PanNETs, accounting for more than 50% of cases, and are clinically important tumors (35, 36, 37). Unlike functioning PanNETs, NF-PanNETs lack hormone-related symptoms, making early detection difficult, and consequently, many patients are not diagnosed until advanced stages. Despite the importance of NF-PanNET in disease, most preclinical studies have focused on functional PanNET models (38). Thus, there has been a critical need to develop reliable NF-PanNET mouse models to clarify the biology of these tumors and to explore potential new therapeutic approaches. We previously established a novel NF-PanNET mouse model and found that a ketogenic diet suppressed insulin signaling, thereby inhibiting the onset and progression of both NF-PanNETs and PitNETs in *Men1*^f/f−^RipCre^+^ mice (9). These findings suggested that glucose control might represent an effective preventive and therapeutic strategy not only for sporadic NF-PanNETs but also for hereditary cases associated with *MEN1* mutations. However, many patients have trouble staying on a ketogenic diet due to dietary preferences and habit. Long-term administration of metformin, a widely prescribed, safe, and well-tolerated anti-diabetic drug, may lessen the need for strict dietary adherence and provide a more practical alternative or adjunct therapy.

In addition to lowering blood glucose, metformin has been proposed to exert antitumor effects through several mechanisms, including AMPK activation, suppression of PI3K/Akt–mTOR signaling, and modulation of the tumor microenvironment (39). Consistent with these reports, our study showed that metformin reduced tumor proliferation, restored p21 expression, and downregulated angiogenesis- and inflammation-related genes, including *Mmp2*, *Mmp9*, and *Nos2*. Given the well-established link between metformin and AMPK activation, we examined phosphorylated AMPK (p-AMPK) levels in PanNET lesions. However, neither immunohistochemistry nor western blotting showed a detectable increase in p-AMPK in metformin-treated mice. These results indicate that direct AMPK-dependent inhibition in tumor cells is unlikely to be the primary mechanism in this setting. Instead, systemic metabolic improvement – such as improved glucose control and restored β-cell glucose responsiveness – likely plays a dominant role in metformin-mediated suppression of NF-PanNET progression in MEN1-associated tumors. However, a limitation of this study is that the mechanistic effects of metformin on β-cell and PanNET proliferation cannot be fully separated from its systemic metabolic actions *in vivo*. Because glucose levels and insulin signaling strongly influence β-cell proliferation (9), it remains difficult to determine whether the observed antiproliferative effects reflect direct tumor-cell-intrinsic mechanisms or secondary consequences of improved metabolic control. Although our data suggest that AMPK activation within tumor lesions is not a dominant mechanism, further *in vitro* studies under controlled metabolic conditions will be required to clarify the direct effects of metformin.

Importantly, our clinical analysis demonstrated that NF-PanNET patients with a history of use of blood glucose-lowering agents had a significantly better OS compared with controls, with no significant differences seen among other clinicopathological factors, such as age, sex, or tumor grade. These findings strongly support the translational relevance of our preclinical mouse model data and suggest that metformin could serve as a clinically meaningful therapeutic adjunct in NF-PanNETs. However, fasting glucose and HbA1c data were not consistently available across all cases in this retrospective cohort, preventing a direct evaluation of how metabolic status may influence treatment response. Future prospective studies including comprehensive metabolic assessment will be essential to validate these clinical implications.

Metformin has shown somewhat mixed outcomes in clinical trials for various cancers (40), and there are reasons to believe that it may be more effective in neuroendocrine tumors. NETs, and particularly NF-PanNETs, are biologically distinct in that they rarely harbor recurrent mutations in canonical oncogenes or tumor suppressors. Instead, their growth is strongly linked to glucose and insulin signaling pathways. This unique biology suggests that NF-PanNETs may be more susceptible to systemic metabolic interventions. By lowering blood glucose and insulin levels and attenuating PI3K/Akt–mTOR signaling, metformin may exert a proportionally greater suppressive effect on NF-PanNETs compared with other tumor types. Our findings in *Men1*^f/f−^RipCre^+^ mice, together with patient survival analysis, provide supportive evidence for this possibility and highlight the need for further clinical evaluation.

Collectively, our findings indicate that regulating blood glucose, through either dietary intervention or pharmacological intervention by drugs such as metformin, may have potential to prevent or treat NF-PanNETs and MEN1-associated NETs. Given that the insulin–PI3K–Akt–mTOR pathway plays a pivotal role in the progression of many tumor types, our study also raises the possibility that metformin therapy could be applicable to a broader range of malignancies beyond neuroendocrine tumors. Future studies should focus on integrating glucose control strategies with early detection approaches and existing therapeutic regimens to develop comprehensive treatment strategies for patients with NF-PanNETs, particularly those with MEN1-associated hereditary predisposition.

## Supplementary materials



## Declaration of interest

The authors declare that there is no conflict of interest that could be perceived as prejudicing the impartiality of the work reported.

## Funding

AN was a JSPS research fellow (2023–2024), and Yu C was a JSPS international research fellow (2020–2022). This study was partly supported by P-Direct, P-Create, and P-Promote from AMED of Japan (RO); P-Promote from AMED of Japan (Yu C); a Grant-in-Aid for Scientific Research (B, #20H03523 and Challenging Exploratory Research, # 23K18382 to RO), a Grant-in-Aid for Early-Career Scientists (#24K19313 to Yu C and #19K16732 to Yu C), a Grant-in-Aid for Research Activity Start-up (#23K19644 to Yu C), a Grant-in-Aid for Scientific Research (C, #22K07181 and #25K13537 to YT) from MEXT of Japan; a Grant-in-Aid for JSPS fellow (#24KJ1825 to AN and #20F20414 to RO and Yu C); and research grants of the Okinaka Memorial Institute for Medical Research (RO), the Life Science Foundation of Japan (RO), Princess Takamatsu Cancer Research Fund (RO), Nagasaki University ‘Doctoral Program for World-leading Innovative and Smart Education’ for Global Health, KYOIKU KENKYU SHIEN KEIHI (AN), Pancreas Research Foundation of Japan (Yu C), Ichiro Kanehara Foundation for the promotion of Medical Sciences and Medical Care (Yu C), HOPE Project Grant for Translational Research (TR) Young Researcher Development Program from Foundation for Promotion of Cancer Research (Yu C), and Young Researcher Grant from Japan Health Research Promotion Bureau (Yu C).

## Author contribution statement

Ohki R conceived and supervised the study. Nakano A, Huang H, Mitsui Y, Shirai R, Chen Y, Tajima T, Sakaguchi Y, Moro T, Hoshino C, Terada K, Sakata N, Yokoyama A, Hijioka S, Tabata Y, and Ohki R performed formal analysis and investigations and designed the methodology. Nakano A, Chen Y, Ishiai M, and Ohki R acquired funding. Nakano A and Ohki R wrote the original draft of the manuscript. Nakano A, Mitsui Y, Shirai R, Chen Y, Tajima T, Sakaguchi Y, Moro T, Sakata N, Yokoyama A, Ishiai M, Kassai H, Tabata Y, and Ohki R reviewed and edited the manuscript.
