# Supplementary figures and images for: Metformin suppresses MEN1-associated pancreatic and pituitary neuroendocrine tumors: evidence from mouse models and clinical data
Source: Endocr Relat Cancer. 2026 Feb 17;33(2):e250518. doi: 10.1530/ERC-25-0518 (PMC12920065; doi:10.1530/ERC-25-0518)

**Fig. S1**

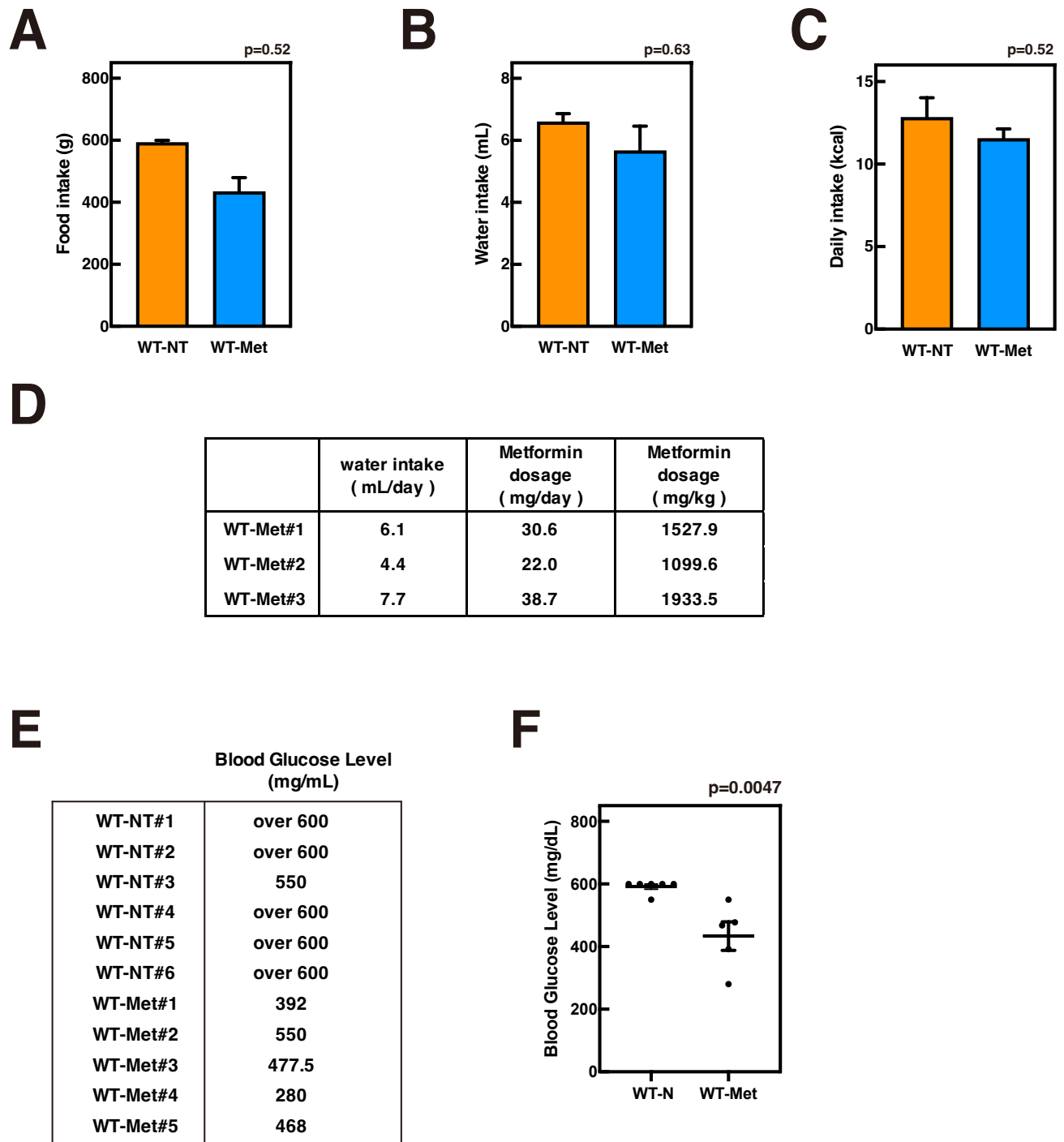

**Fig. S2**

**A**

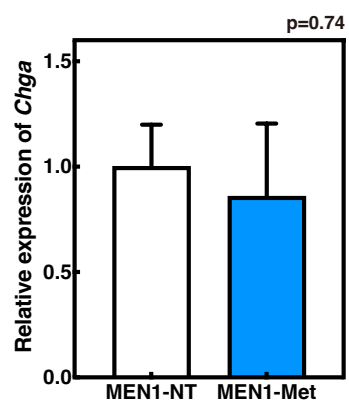

**B**

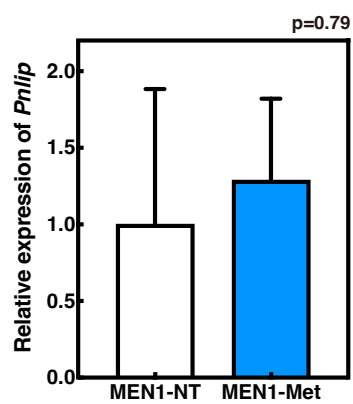

Supplement: Supplementary file 1 [file supplementary_materials.pdf]
